# Impact of Stacking-Up and Scaling-Down Bit Cells in 3D NAND on Their Threshold Voltages

**DOI:** 10.3390/mi13071139

**Published:** 2022-07-18

**Authors:** Dongwoo Lee, Changhwan Shin

**Affiliations:** 1 Department of Semiconductor and Display Engineering, Sungkyunkwan University, Suwon 16419, Korea; chobokyi@hanmail.net; 2 School of Electrical Engineering, Korea University, Seoul 02841, Korea

**Keywords:** 3D NAND flash, nanowire channel, macaroni channel, tapered channel

## Abstract

Over the past few decades, NAND flash memory has advanced with exponentially-increasing bit growth. As bit cells in 3D NAND flash memory are stacked up and scaled down together, some potential challenges should be investigated. In order to reasonably predict those challenges, a TCAD (technology computer-aided design) simulation for 3D NAND structure in mass production has been run. By aggressively stacking-up and scaling-down bit cells in a string, the structure of channel hole was varied from a macaroni to nanowire. This causes the threshold voltage difference (ΔV_th_) between the top cell and bottom cell in the same string. In detail, ΔV_th_ between the top cell and bottom cell mostly depends on the xy-scaling, but the way how ΔV_th_ is affected is not very dependent on the stack height.

## 1. Introduction

**Figure 1:**
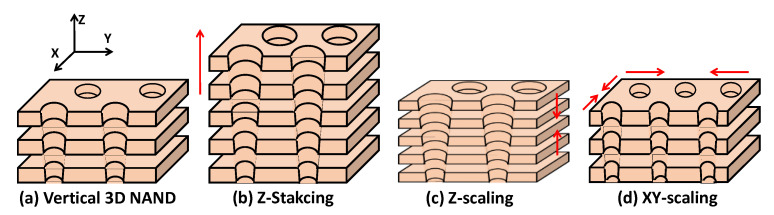
(**a**) 3D bird’s-eye view of vertical 3D NAND flash memory. There exist three ways to increase the memory density, i.e., (**b**) increasing the number of stacks in z-direction, (**c**) decreasing the thickness of each stack, and (**d**) decreasing the cell-to-cell pitch in the xy-plane.

Over the past decade, NAND flash memory technology has been transformed from 2D NAND to 3D NAND. As of today, the 8th generation of 3D NAND (i.e., the number of NAND bit cells in a string >200 stack) is about to be ready for mass production. There still exists some technical challenges for next-generation 3D NAND products [1], such as retention issue originated from charge trap layer, coupling issue by floating channel structure, and/or manufacturing TAT (turn-around-time) by process complexity, etc. Among them, the device design for achieving higher memory density becomes trickier. In other words, the physical size of NAND bit cell should be aggressively scaled down for higher memory density with better performance and less power consumption as well as cost effectiveness [2]. When it comes to scaling down the physical dimension of a 3D NAND cell, three possible approaches, i.e., z-stacking, z-direction scaling, and xy-direction scaling, would be available (see Figure 1) [1]. Showing off how advanced the 3D NAND technology has been, the z-stacking has been the conspicuous approach for implementing high memory density. With the help of the z-direction scaling, the total height of a string was not increased as much as the number of vertical wordlines. In reality, the height of vertical channel hole has increased by 1.5 times, while the number of vertical wordlines (i.e., the number of 3D NAND bit cells in a string) has increased by two times [3]. It is expected that the pitch between two neighboring channel holes becomes widened, as long as the vertical channel hole is slightly tilted. However, the pitch along the xy-direction was not increased with the help of improved dry etching techniques as well as the adoption of double-deck etching scheme (i.e., two separate etching steps for a single channel hole) [3].

With the three different approaches of xy-/z-scaling and z-stacking for higher memory capacity discussed above in mind, it is logical to bring up nanowire structure as a technical question related to the shape of the channel hole in 3D NAND (not of macaroni, which is the current structure of the channel hole in 3D NAND). In this work, a tectonic shift in the channel hole of 3D NAND is investigated. Some potential assumptions on how the electrical characteristics of 3D NAND are affected are to be provided/reviewed, and then, a few scenarios for xy-scaling and z-stacking are simulated for quantitative analysis.

## 2. Potential Challenges in 3D NAND Structure

**Figure 2:**
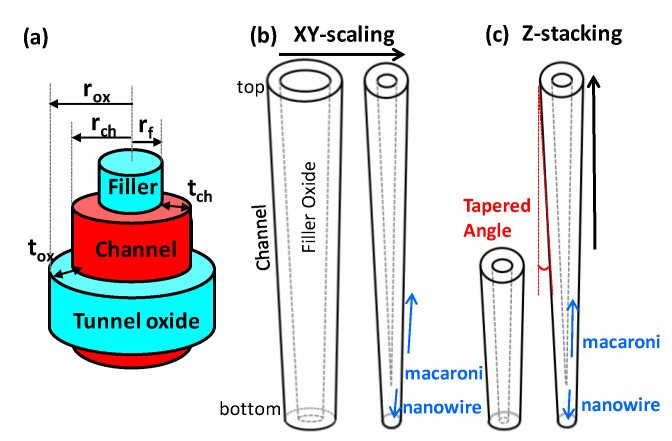
(**a**) Illustrated macaroni structure of 3D NAND. Schematic of showing how the shape of a channel hole in 3D NAND is varied by (**b**) XY-scaling, (**c**) Z-stacking. Note that the cells at/near the bottom would have a nanowire structure because of the tapered structure of the channel hole.

A Gate-all-around (GAA) device structure has been adopted for NAND Flash memory device, so that the channel region was surrounded by gate oxide (more specifically, tunnel oxide). In addition, a macaroni device structure in the 3D NAND bit cell was used/formed because of the oxide filler (see Figure 2a). Taking into account the device structure in 3D NAND, the shape of the channel hole would evolve from a macaroni into a nanowire, in the end. In this work, two cases for the shape of the channel hole are investigated, one is associated with xy-direction scaling, and the other is with z-stacking. With the xy-direction scaling, the radius of the channel hole at the top and bottom cells would be scaled down together (see Figure 2b). However, because of a certain tapered angle, the radius of the bottom cell in a string should become shorter than the radius of the top cell in the same string (see Figure 2c). When considering together both xy-direction scaling and z-stacking, it is likely for the channel hole to become a nanowire.

One of the main motivations of adopting a macaroni-shaped channel hole in 3D NAND is associated with the trap density at the grain boundary of the poly-silicon channel material [4]. A thin poly-silicon layer in the channel hole forms a fully depleted channel region, and therefore, the subthreshold characteristics of 3D NAND bit cell becomes less sensitive to the trap density in the grain boundary. With the adoption of the macaroni-shaped channel structure, there exist a few more merits from the perspective of device operation, e.g., steeper sub-threshold swing, narrow distribution of the drain current, and more robustness to the threshold voltage variation. Note that the macaroni-shaped channel structure (vs. non-macaroni or nanowire channel structure) provides us with a much thinner channel layer and, thereby, a lower number of grain boundaries in the channel [5]. This would be the main reasons for the merits above.

Because of the unavoidable tapered angle in the channel hole, the channel diameter of top bit cell is different from that of bottom bit cell (Figure 2). This would induce a non-uniform electric field intensity along the z-direction [6,7]. In detail, the electric field intensity of the bottom bit cell with a shorter channel diameter becomes stronger, and thereby, its threshold voltage is lower/higher than that of the top cell, depending on the tapered angle of channel hole [7,8]. If the channel diameter of the bottom bit cell aggressively decreases (e.g., below a certain critical value), the threshold voltage of the bottom bit cell would be affected due to the variation of the internal potential profile [9,10].

Another potential challenge in 3D NAND structure would be primarily originated from an interface trap. There exist various types of interface traps at the Si/SiO_2_ interface between the filler oxide and channel as well as between the tunnel oxide and channel (see Figure 2a). These traps would potentially affect the threshold voltage modulation of the bit cells in a string [11]. It is straightforward that a shorter channel diameter of the bottom bit cell brings less amount of interface traps at the filler oxide/channel interface area, because the radius of the filler oxide physically decreases. Especially, if the channel structure of the bottom bit cell becomes a nanowire or similar, the interface traps would be significantly decreased. This structure-induced variation in the amount of interface traps in 3D NAND should cause the threshold voltage variation of the bit cells for a given string.

Subthreshold slope (SS) of each bit cell would be affected by the variation of r_f_ and r_ox_ (see Figure 2a). SS can be physically modeled and be proportional to the ratio of depletion layer capacitance (C_dm_) to oxide capacitance (C_ox_), as in Equation (1) [12].

(1) $$\mathrm{SS}=2.3\frac{\mathrm{kT}}{q}\;\left(1+C_{\mathrm{dm}}/C_{\mathrm{ox}}\;\right)$$

As long as the channel structure looks like a macaroni and the channel thickness (t_ch_) is thin enough to become fully depleted [4,13], C_dm_ and C_ox_ can be simply described by the cylindrical capacitor model in Equation (2) [14].

(2) $$C_{\mathrm{dm}}=\frac{2{\pi \varepsilon }_{\mathrm{Si}}L_{g}}{\ln\left(r_{\mathrm{ch}}/r_{f}\right)}\left({\mathrm{when}\;r}_{f}>0\right)\;C_{\mathrm{ox}}=\frac{2{\pi \varepsilon }_{\mathrm{ox}}L_{g}}{\ln\left(r_{\mathrm{ox}}/r_{\mathrm{ch}}\right)}\left({\mathrm{when}\;r}_{f}>0\right)$$

From Equations (1) and (2), the equation for SS can be re-written as shown in Equation (3).

(3) $$\mathrm{SS}=2.3\frac{\mathrm{kT}}{q}\left(1+\frac{\varepsilon_{\mathrm{Si}}}{\varepsilon_{\mathrm{ox}}}\times \alpha \right),\;\alpha =\frac{\ln\left(r_{\mathrm{ox}}/r_{\mathrm{ch}}\right)}{\ln\left(r_{\mathrm{ch}}/r_{f}\right)}$$

Note that, with r_ch_, r_ox_ and r_f_ can be expressed with t_ox_ and t_ch_, respectively, as shown in Equation (4).

(4) $$r_{\mathrm{ox}}=r_{\mathrm{ch}}+t_{\mathrm{ox}}\;r_{f}=r_{\mathrm{ch}}-t_{\mathrm{ch}}$$

From Equations (3) and (4), we can derive Equation (5).

(5) $$\alpha =\frac{\ln\left(1+t_{\mathrm{ox}}/r_{\mathrm{ch}}\right)}{\ln\left(r_{\mathrm{ch}}/\left(r_{\mathrm{ch}}-t_{\mathrm{ch}}\right)\right)}$$

**Figure 3:**
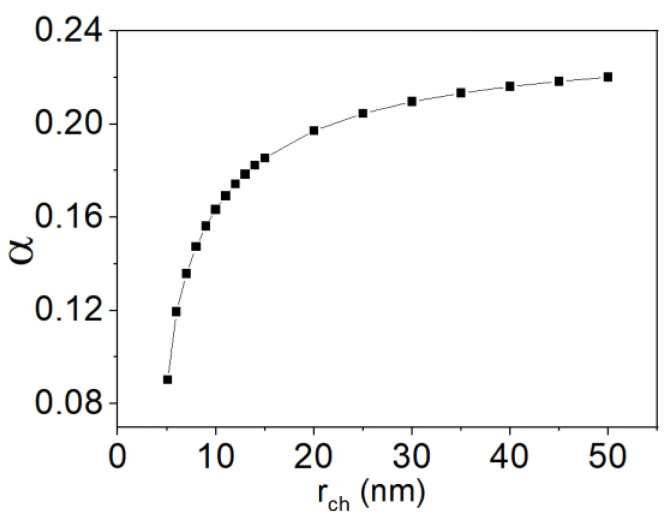
Plot of α (in Equation (3) for SS) vs. r_ch_, when both t_ox_ and t_ch_ is 5 nm. Note that α is significantly decreased if r_ch_ is 15 nm or shorter.

Herein, t_ox_ and t_ch_ are set to 5 nm. Then, a plot of α (in Equation (3) for SS) vs. r_ch_ can be obtained (see Figure 3). As shown in Figure 3, the channel radius of 15 nm or shorter would result in a significant modulation in the capacitance ratio between C_dm_ and C_ox_. This should cause a significant variation of SS.

## 3. Details on Simulation Set-Up

**Figure 4:**
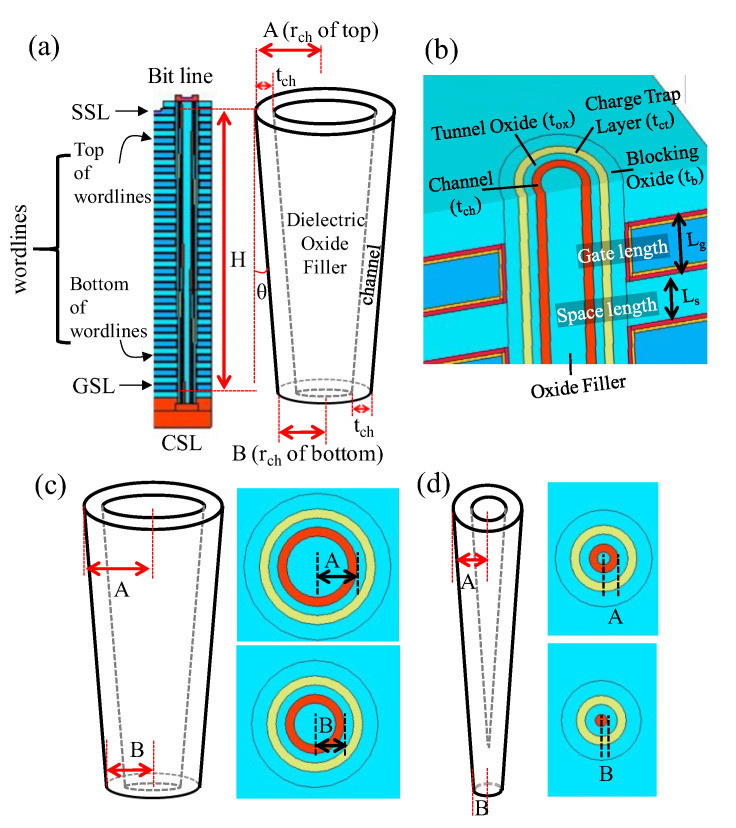
(**a**) Cross-sectional view of the 3D NAND structure along the z-direction. (**b**) 3D bird’s-eye view of the 3D NAND structure. (**c**) Cross-sectional view of the non-aggressively-scaled-down channel hole in the xy-plane at top/bottom WL. (**d**) Cross-sectional view of the very-aggressively-scaled-down channel hole in the xy-plane at top/bottom WL. In the very-aggressively-scaled-down channel hole of the 3D NAND structure, A and B decrease due to the tapered shape of the channel hole.

Using the Synopsys Sentaurus technology computer-aided design (TCAD) tool, the impact of xy-scaling and z-stacking on the electrical characteristics of 3D NAND cells in a string are evaluated. Based on practical design rules for mass production in industry, tapered angle (θ), gate length (L_g_), space length (L_s_), blocking oxide layer thickness (t_b_), charge trap layer thickness (t_ct_), and tunnel oxide layer thickness (t_ox_) are set as 0.3°, 29.0 nm, 22.0 nm, 8.5 nm, 5.0 nm, and 5.0 nm, respectively (Figure 4a,b). The channel thickness (t_ch_) is set/limited to 5.0 nm, if the diameter is long enough (Figure 4c). However, once the radius of the bottom cell (r_ch_, B) becomes smaller than 5.0 nm by xy-scaling and by the tapered angle (θ), the channel structure becomes a nanowire (Figure 4d).

In the simulation, two parameters, i.e., the channel radius and vertical channel height were varied. The top channel radius (A) was varied from 56.5 nm to 9.0 nm, and the bottom channel radius (B) was varied from 51.5 nm to 4.0 nm. The vertical channel height (H) was nominally set to 1 µm. To see the impact of z-stacking, H was increased up to 3 µm by 1µm (i.e., H = 2 µm, and H = 3 µm).

The voltage for the erase operation (V_erase_) was set as 20 V (which is currently used in industry). For the read operation, the voltage applied to a bit line was set to 1 V. In addition, the common source line (CSL) was grounded. The voltage for selected wordlines was varied in-between 0 V and 1 V. Note that the other unselected wordlines were set to V_pass_, to turn them on.

## 4. Results and Discussion

**Figure 5:**
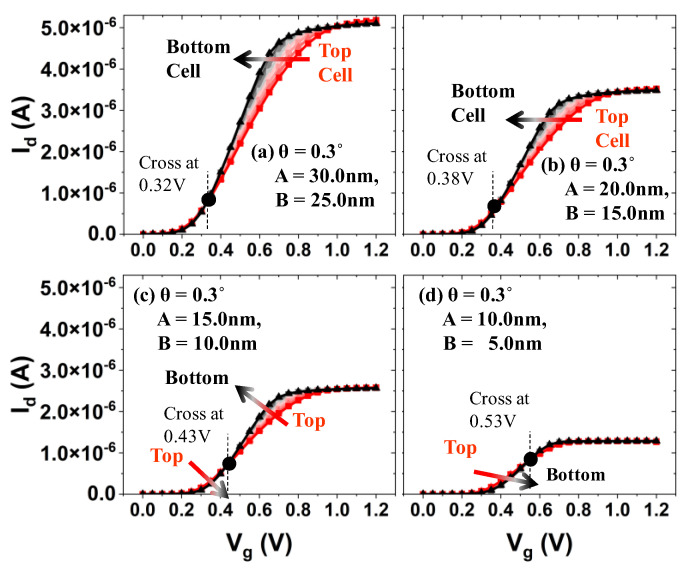
(**a**–**d**) Simulated I_d_ vs. V_g_ for the bit cells (from top to bottom), when those cells in a string are scaled down together in XY-direction.

**Figure 6:**
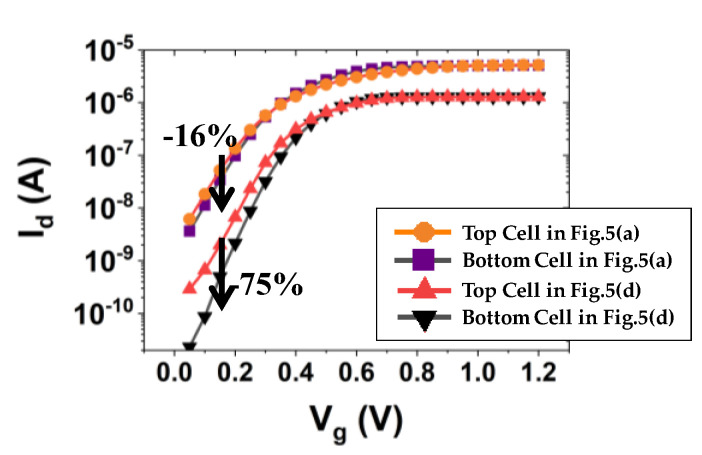
Simulated I_d_ vs. V_g_ to explicitly show the subthreshold characteristics of top/bottom cells, depending on the physical dimensions of the channel hole structure. Note that the subthreshold swing was improved with the smallest physical structure of cells, and its drain current was exponentially affected (increased/decreased).

Simulated I_d_ vs. V_g_ for bit cells in a string is shown in Figure 5. Note that the radius of the channel in each bit cell is not identical due to the tapered structure of the channel hole (i.e., the top bit cell has the longest radius of the channel). For the given tapered angle (θ) and vertical height (H) of the channel hole (i.e., θ = 0.3°, H = 1 µm), the top channel radius (A) and bottom channel radius (B) are varied (or scaled down in xy-directions, as shown in Figure 5a–d). It turned out that the bottom bit cell has superior switching characteristic, i.e., a lower off-state leakage current (see Figure 6), higher on-state drive current (see Figure 5a), and comparable threshold voltage (see Figure 6) (notice that, if the threshold voltage is determined by the constant current method, the threshold voltage of the bottom cell is slightly higher than that of the top cell). Moreover, as the physical dimension of the channel hole is aggressively scaled down in xy-directions (see from Figure 5a–d), the wordline voltage at which a comparable channel current is achieved in both top and bottom cells becomes higher (see the cross-over point in Figure 5a–d).

**Figure 7:**
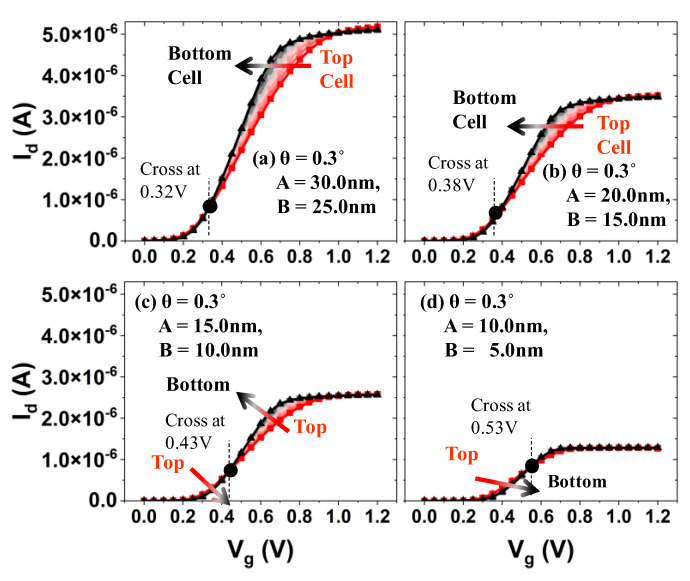
Simulated I_d_ vs. V_g_. with xy-scaling, the subthreshold swing (SS) improves in both (**a**) bottom cell and (**b**) top cell. When the form of the channel near or at the bottom cell looks like a nanowire structure (i.e., A = 10.0 nm, B = 5.0 nm), SS becomes 77.5 mV/dec at 300 K.

For a few combinations of A and B (determined by scaling in xy-directions), Figure 7 shows the simulated input transfer characteristics of the bottom cell and top cell. For all the combinations, the subthreshold swing of bottom cells (Figure 7a) is better than that of top cells (Figure 7b). As a result, for an identical scaling in xy-directions (of course, with constant tapered angle in this work), the input transfer characteristics of the bottom cell is more varied than that of the top cell (see the black-colored arrow in Figure 7).

**Figure 8:**
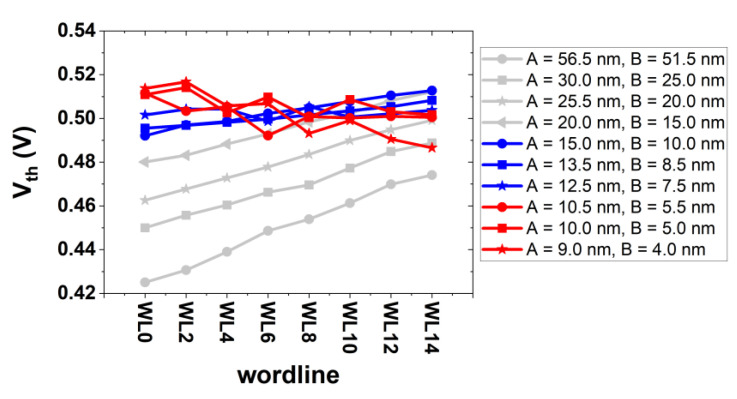
Estimated threshold voltage of bit cells from the bottom side (w0) to the top side (w14) for a given string. Depending on the channel radius (A), the threshold voltage (V_th_) of the bottom cell is either higher or lower than V_th_ of the top cell (e.g., for A = 20.0~56.5 nm (for A = 9.0~10.5 nm), V_th_ of the bottom cell is lower (higher) than V_th_ of the top cell). The non-monotonic decrease of V_th_ for all bit-cells with A of 10.0 nm can be understood due to the peaked-out value of the eDensity for a few bit-cells placed in the middle of the string.

Depending on the physical dimension of the channel radius, the threshold voltage of each bit cell in a string is different from that of the others (see Figure 8): (i) for the range of (A, B) = (20.0 nm, 15.0 nm)~(56.5 nm, 51.5 nm), the bottom cell in the vertical string has a lower threshold voltage than the top cell. (ii) For the range of (A, B) = (12.5 nm, 7.5 nm)~(15.0 nm, 10.0 nm), the threshold voltage of top and bottom cell becomes comparable. (iii) For the range of (A, B) = (9.0 nm, 4.0 nm)~(10.5 nm, 5.5 nm) (herein, the bottom cell has a nanowire structure), the bottom cell has a higher threshold voltage than the top cell.

**Figure 9:**
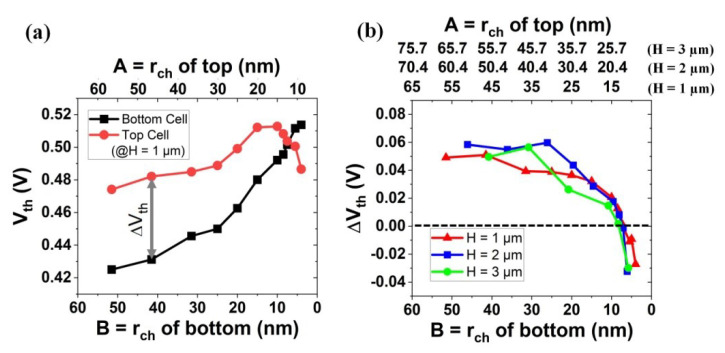
(**a**) Estimated threshold voltage (V_th_) for various channel diameters. Note that V_th_ for the top cell is higher (lower) than that for the bottom cell, if the channel radius of bottom cell (B) is longer (shorter) than ~10 nm. (**b**) Threshold voltage difference vs. channel radius, for a few given channel heights, i.e., 1 µm, 2 µm, and 3 µm. ΔV_th_ is quite comparable, although the height becomes doubled or tripled. Note that the tapered angle, θ, is set to 0.3°.

In Figure 9a, the estimated threshold voltage for various channel diameters is summarized. Note that the bottom channel radius (B) of 3D NAND in mass production is in the range of 40~50 nm [3]. By the xy-direction scaling, the threshold voltage of the top cell and bottom cell slightly increases, if B is > 20 nm. However, if B is 10 nm or below, the threshold voltage of the top cell becomes lower (notice that, if the threshold voltage is determined by the constant current method, the threshold voltage of the top cell becomes relatively lower than that of the bottom cell; see Figure 5). In Figure 9b, by the xy-scaling, the threshold voltage difference (ΔV_th_) between the top cell and bottom cell is summarized. Moreover, in order to figure out the impact of z-stacking on ΔV_th_, ΔV_th_ for a higher H (i.e., 2 µm or 3 µm instead of 1 µm) was included in Figure 9b. It turned out that the way how ΔV_th_ is varied by xy-scaling was not significantly affected by the height of the stack (H).

## 5. Conclusions

To address technical challenges for next-generation 3D NAND products, a few scenarios for xy-scaling and z-stacking were quantitatively evaluated. Because of the unavoidable tapered angle in the channel hole, the shape of channel hole would evolve from a macaroni into a nanowire. This should cause a few challenges, i.e., the variation of internal potential profile, the amount of interface traps at the filler oxide/channel interface area, and the undesirable ratio of depletion layer capacitance to oxide capacitance. Due to the physical dimension of channel radius, the threshold voltage of each bit cell in a string is different from that of the others. It turned out that the threshold voltage difference (ΔV_th_) between top cell and bottom cell depends on the xy-scaling. In detail, for given θ = 0.3°, H = 1 µm, in the range of (A, B) = (20.0 nm, 15.0 nm)~(56.5 nm, 51.5 nm), the bottom cell in string has a lower threshold voltage than the top cell. However, in the range of (A, B) = (9.0 nm, 4.0 nm)~(10.5 nm, 5.5 nm), the bottom cell has a higher threshold voltage than the top cell. On the other hand, the way how ΔV_th_ is varied by the height of the stack (H) is not significant.

## Data Availability

The data presented in this study are available on request from the corresponding author.
